# Magnetoelectric interaction in molecular multiferroic nanocomposites†

**DOI:** 10.1039/d2ra04060c

**Published:** 2022-08-24

**Authors:** Alireza Jalouli, Shenqiang Ren

**Affiliations:** Department of Mechanical and Aerospace Engineering, University at Buffalo, The State University of New York Buffalo New York 14260 USA alirezaj@buffalo.edu; Department of Chemistry, University at Buffalo, The State University of New York Buffalo New York 14260 USA; Research and Education in Energy Environment & Water Institute, University at Buffalo, The State University of New York Buffalo New York 14260 USA

## Abstract

Incorporation of magnetic and electric orders in a form of multiferroics is an interesting topic in materials science. Making a molecular heterogeneous composite by incorporating the molecular magnet vanadium–chromium Prussian blue analogue (V–Cr PBA) and a molecular ferroelectric imidazolium chloride C_3_N_2_H_5_-ClO_4_ (ImClO_4_) provides a pathway towards achieving the room temperature magnetoelectric effect. The change of magnetization of about 6% is shown as a result of applying an electric field (21 kV cm^−1^) to the composite made of the aforementioned molecular crystals at room temperature. In the ferromagnetic resonance measurement (FMR) under the effect of an applied electric field, a shift of the resonance magnetic field is also observed in the nanocomposites. This work provides a pathway towards molecular multiferroic nanocomposites with magnetoelectric coupling interactions at room temperature.

## Abstract 2

Incorporation of molecular magnetic and ferroelectric V–Cr PBA and ImClO_4_ introduces a room temperature multiferroic composite.

## Graphical abstract

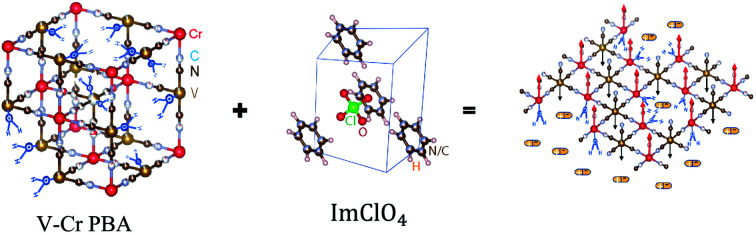

## Introduction

The search for multifunctional materials with coupling effects between electric and magnetic properties is an interesting topic in materials engineering. However, in the realm of molecular chemistry and physics, there is an equal interest in exploring molecular structures that can be tuned or respond to external stimuli manipulations. In that respect, multiferroic materials that simultaneously exhibit both electrical and magnetic properties are an active field of research.^1^ Some of the potential applications of multiferroic materials include microwave devices,^2,3^ energy harvesting, and memory storage devices.^4,5^ Molecular ferroic materials^6,7^ have the flexibility of incorporating their structures^8^ for exploring new compositions that demonstrate coupling between electric and magnetic responses.^9–11^

One of the challenges in the path of achieving molecular multiferroic is incorporating suitable ferromagnetic and ferroelectric materials together in such a way that both phases exist simultaneously under the specific temperature, particularly room-temperature multiferroic orders. One of the promising molecular ferrimagnet families are Prussian blue analogues (PBA) with general formula M_A_(M_B_(CN)_6_)_b_. *n*H_2_O (M_A_ M_B_ = transition metals, CN = cyanide ligand).^12^ In this context, vanadium–chromium PBA (V–Cr PBA) molecular ferrimagnets show room temperature magnetic ordering transition.^6,13,14^ In Prussian blue analogues V–Cr PBA, the water molecules coordinate with vanadium cations to compensate the vacancies in the PBA lattice. This create zeolitic water molecules existing in the PB lattice,^15^ leading to the formation of hydrogen bonding networks that are capable to take part in a charge transfer mechanism of proton transfer.^16^ On the other hand, the water molecules are also connected with some of the vanadium atoms which by themselves are responsible for the magnetic properties of PBA. There are two promising strategies to change the magnetic properties of a molecular magnet material: the pressure and electric field stimuli.^10,17^ In some PBA, the pressure up to 0.8 GPa can shift magnetization up to 10%, at temperatures well below the transition temperature.^17^ In both scenarios, the stretching in the M_A_^II^=C≡N=M_B_^III^ structure is the responsible for the change of symmetry and the modification of the magnetic property consequently.

In this work, room temperature molecular ferroelectric crystal ImClO_4_^18^ is selected with V–Cr PBA magnet for the preparation of heterogeneous composites.

At room temperature, ImClO_4_ phase exhibits high polarization of 8 μC cm^−1^.^19,20^

In the 1 : 1 ratio between ImClO_4_ and V–Cr PBA, we demonstrate a noticeable response at room temperature with six percent of magnetization tunability when the bias electric field is in the order of 20 kV cm^−1^.

## Main text

### Material characterization

**Fig. 1:**
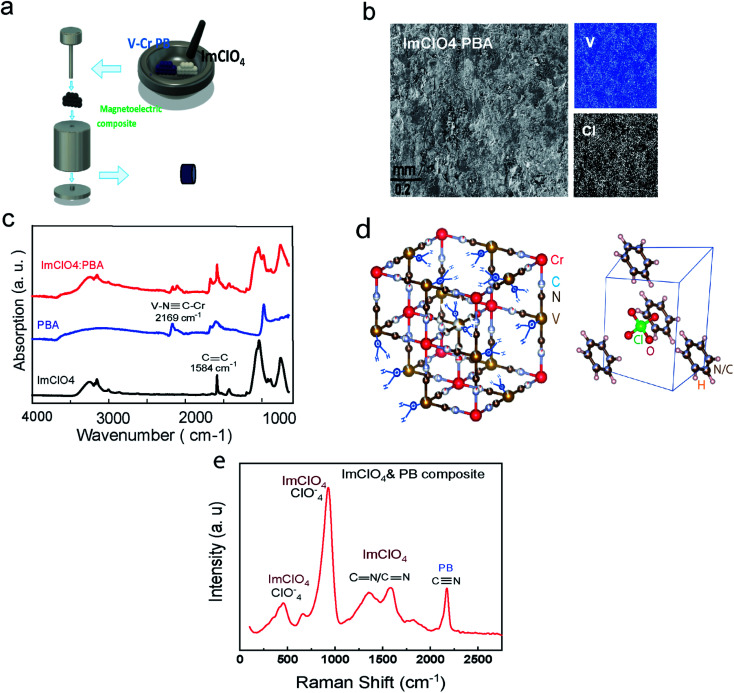
(a) The process of ImClO_4_ : PBA preparation. (b) SEM/EDS scan of an area on the composite shows an even distribution of the elements of each of the constituents (vanadium as the representative of V–Cr PBA and Cl for ImClO_4_ crystals are positive and negative photos). (c) FTIR spectra of ImClO_4_. PB, and the composite are displayed; the C=C mode in ImClO_4_ and N=C in PB are not changing the composite of the mixture. (d) V–Cr PB crystal with vacancies and the water molecules that randomly connected to vanadium atoms and the ImClO_4_ crystal at room temperature. (e) Raman shift spectrum of the composite ImClO_4_ and PB with the signature peaks of each.

Magnetic V–Cr PBA and ferroelectric ImClO_4_ crystal structure are displayed in Fig. 1(d). In crystal phase, ImClO_4_ exhibits a hexagonal symmetry in contrast to the five-member ring symmetry of its molecular structure.^18,21^

To prepare PB powder crystals, the power of potassium hexacyanochromate(iii) and vanadium(ii) chloride are mixed with 3 : 2 weight ratio in water and is left in a centrifuge tube for one day to react completely. The product is washed for a few times and then centrifuged to remove the unreacted agents. The achieved substance with a dark blue color is dried for one day in a vacuum chamber at room temperature.^7,22^ The product is a dark blue powder of PB nanocrystals. Since the PB powder is sensitive to oxidation, it is necessary to be kept in the glove box. To synthesize ImClO_4_, equal molar amounts of imidazolium (C_3_N_2_H_4_) with perchloric acid (HClO_4_) are mixed. The solvent is dried slowly in several days to achieve a white transparent powder crystal.^18^ Different ratios of the synthesized crystals (PB and ImClO_4_) were ground together and then pressed to have round palettes with 6 mm diameter and about 1 mm thickness. To contact a thin copper wire to the top and bottom of the composite palette sample, silver epoxy was used (Fig. 1(a), S1†). The Scanning electron Microscopy (SEM) along with the elements scanning of the composite (Fig. 1(b), S1†) show an even distribution of Cl atoms as representative of ImClO_4_ and V atoms as exist in V–Cr PBA component. The HitachiSU70 SEM/EDS microscope was used for the surface image and also elements scan. In Fig. 1(e), the Raman shift for in-plane-deformation, symmetric and antisymmetric stretching modes of ClO_4_^−^ at 669 cm^−1^, 929 cm^−1^ and 1069 cm^−1^ are depicted. The other peak depicted is assigned to C=C/C=N symmetric stretching at 1448, 1586 cm^−1^ respectively. The Raman spectra system, Renishaw with diode laser (488 nm), was used for the previous part.

In order to confirm the presence of both materials (PBA and ImClO_4_) in the composite, we measured the Fourier-Transform infrared (FTIR) spectra of each of the constituents before mixing and the as-produced composite.

In Fig. 1(c), the FTIR spectra of the ferroelectric ImClO_4_, PBA, and the composition of them is displayed. The CC absorption in ImClO_4_ near 1600 cm^−1^ is displayed.^23^ In V–Cr PBA, the peaks located at 2113 and 2169 are assigned to stretching bonds, V(ii)–N≡C–Cr(iii) and, V^IV^O–N≡C–Cr(iii) respectively.^6,12^ The presence of V^IV^=O with the assigned peak at 981 cm^−1^ is due to being exposed to air for a short time during the synthesis process.^6^ The ratio of V(iii) to V(ii) can have a slight shift in *T*_C_ ranges between 296 to 310 ± 8 K. This will not be very significant change for the suggested properties here. The Agilent Cary 630 FTIR spectrometer which is suitable for dry-powder samples was used for the previous part.

### Magnetoelectric coupling

**Fig. 2:**
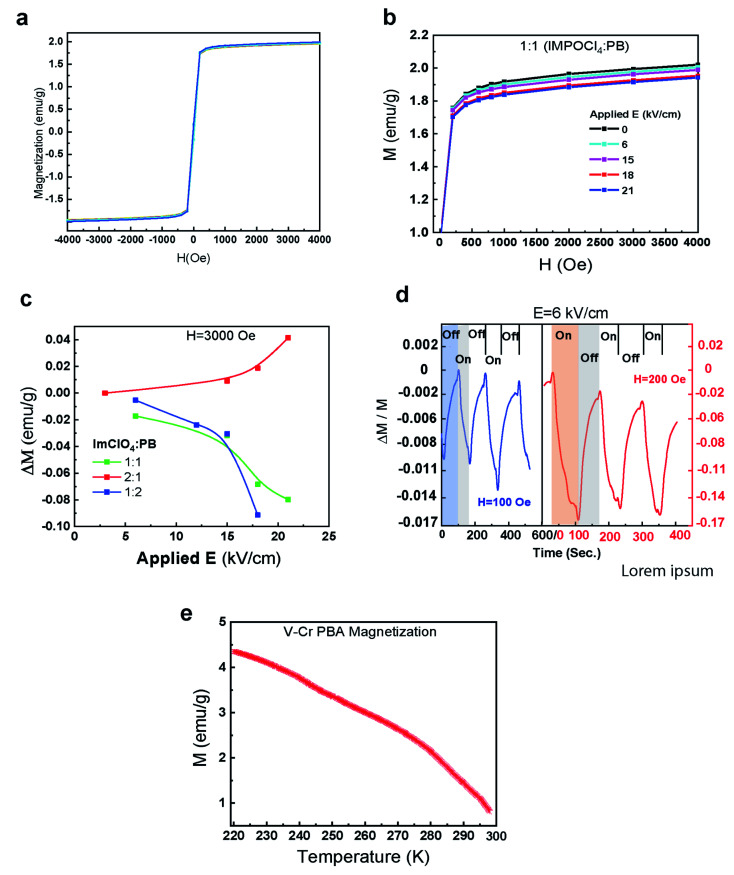
(a) and (b) the whole hysteresis loop of 1 : 1 is displayed (b) the half of the hysteresis loop of 1 : 1 ratio and the splitting at 0, 6, 15, 18, and 21. (c) The change of magnetization at the saturation part for 1 : 1,1 : 2, and 2 : 1 at different Es. (d) The effect of turning on/off of the applied electric field at H = 100 and 200 Oe are displayed for 1 : 1 at *E* = 6 kV cm^−1^. (e) The magnetization *vs.* temperature for a V–Cr PBA.

The interplay between the applied electric field and magnetization of PBA-ImClO_4_ show the splitting of the saturation part of magnetization hysteresis curves. In the Fig. 2(a), the magnetization of such composites can experience a change of about 6% as a result of increasing the electric field from 0 to 21 (kV cm^−1^). The Microsense EZ7-380V vibrational sample magnetometer (VSM) was used to measure room-temperature magnetization as well as magnetization *versus* temperature. The composition ratio between PBA and ImClO_4_ plays an important role in the magnitude of the magnetization change. In Fig. 2(b), the magnetic response to the applied electric field in 1 : 1 composite at two different magnetic field biases is demonstrated. The amplitude of the response increases by increasing the magnetic field bias. The relative magnetization at *H* = 100 and 200 Oe changes 0.011 and 0.017 respectively under the on/off cycle of electric field bias *E* = 6 kV cm^−1^. It is observed that 2 : 1 in contrast to 1 : 1 and 1 : 2 ratios, exhibit an opposite magnetic behavior under the electric field biases (Fig. 2(c), S2†).

The magneto-electric interaction can be further investigated through FMR.^24^ The FMR shift could be resulted from the E-field induced change of magnetic symmetry of the structure.^25^

**Fig. 3:**
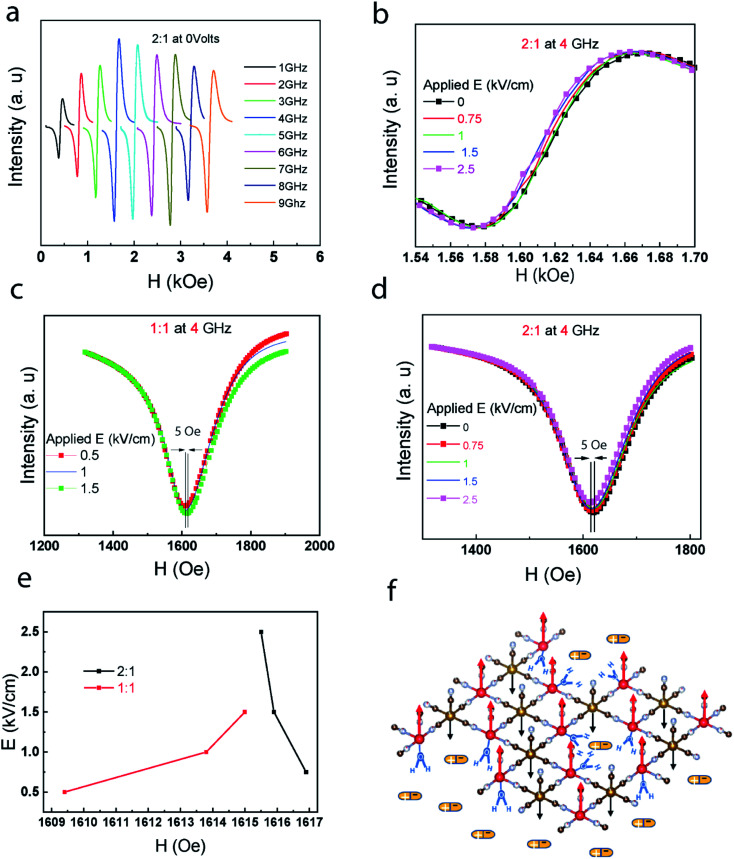
(a) FMR spectra of the 2 : 1 for various frequencies (different H) at *E* = 0. (b) The FMR signal shift for 2 : 1 at 4 GHz for the applied Electric fields ranging from 0 to 2.4 kV cm^−1^. (c) and (d) Integrated FMR spectra of 2 : 1 and 1 : 1 at 4 GHz for different electric biases. (e) Resonance peak shift as a function of *E* for 1 : 1 and 2 : 1. (f) Vanadium and chromium atoms with their spins oriented in opposite directions (ferrimagnetic) and the ImClO_4_ as electric dipoles in contact with PBA layer.

In the absence of an external electric field, the change of the resonance frequency of composites as a result of the applied magnetic field intensity shows a similar trend regardless of the ratio compositions (Fig. 3(a) and S3†). In the FMR measurement, the magneto-electric coupling is tested at specific frequencies for the composites. In Fig. 3(b), there is an asymmetrical shift in 2 : 1 FMR curve at 4 GHz. The shift in the position of the resonance field is more recognizable in the integrated curve of the FMR (Fig. 3(c) and (d)). These confirms the same opposite patterns of magnetic response to the electric bias that was shown in the VSM section (Fig. 3(e)).

In Fig. 3(f), one of the suggested mechanisms based on proton-transfer mechanism is demonstrated. The applied electric field through the electric dipoles of the ferroelectric molecules of ImClO_4_ influence protons connected to the vanadium atoms. The electric field manipulation to this collective spin–spin interaction^26^ can lead to the FMR frequency shifts. To explain the opposite pattern observed in 1 : 1 compared to 2 : 1, there is a possibility here. While the spin alignment is enhanced in 2 : 1 due to the strained is applied by ImClO_4_ dipoles, this might be detrimental to the spin alignment in 1 : 1 ratio. To run the FMR measurement we used a home-made setup. It is quite similar to magnetic-resonance system such as NMR and EPR. The principle is based on applying a constant magnetic field to make the ferromagnetic regions undergo precession with Larmor frequencies proportional to the applied H.

The alternative magnetic field which is perpendicular to the constant magnetic field orientation will provide the resonance condition if the frequencies match is met.

## Conclusions

Prussian blue analogues are molecular ferrimagnetic insulator with a bandgap 3 eV and conductivity of 10^−3^ S cm^−1^.^16,27^ It has not been reported of sign of electric field effect on bulk material of PBAs to date. Room-temperature molecular multiferroic nanocomposites comprised of molecular ferromagnet V–Cr PBA and ferroelectric ImClO_4_ suggest a pathway to incorporate magnetic and electric coupling effects at room temperature. The molecular structure of the constituents and the capability of changing the ratio allow us to tailor the characteristics in order to achieve on-demand magnetoelectric properties. The change of magnetization for various ratios of ImClO_4_ : PBA can lead to the change of magnetization up to about 6% under the electric field bias. The FMR measurement shows a slight shift of resonance position by applying a relatively weaker electric field (6 kV cm^−1^).

## Author contributions

A. Jalouli conducted the project and wrote the manuscript. Dr S. Ren designed the project.

## Conflicts of interest

There are no conflicts to declare.

## Supplementary Material

RA-012-D2RA04060C-s001

## References

[cit1] Spaldin N. A. (2020). Proc. Math. Phys. Eng. Sci..

[cit2] Jalouli A., Khuje S., Sheng A., Islam A., Di Luigi M., Petit D., Li Z., Zhuang C.-G., Kester L., Armstrong J., Yu J., Ren S. (2021). ACS Appl. Nano Mater..

[cit3] Li C., Khuje S., Petit D., Huang Y., Sheng A., An L., Di Luigi M., Jalouli A., Navarro M., Islam A., Ren S. (2021). Nanotechnology.

[cit4] Vopson M. M. (2015). Crit. Rev. Solid State Mater. Sci..

[cit5] Spaldin N. A., Ramesh R. (2019). Nat. Mater..

[cit6] Garde R., Villain F., Verdaguer M. (2002). Journal of the American Chemical Society.

[cit7] Hu Y., Broderick S., Guo Z., N'Diaye A. T., Bola J. S., Malissa H., Li C., Zhang Q., Huang Y., Jia Q., Boehme C., Vardeny Z. V., Zhou C., Ren S. (2021). Nat. Commun..

[cit8] Pardo E., Train C., Liu H., Chamoreau L. M., Dkhil B., Boubekeur K., Lloret F., Nakatani K., Tokoro H., Ohkoshi S., Verdaguer M. (2012). Angew. Chem., Int. Ed. Engl..

[cit9] Liu W., Osanloo M. R., Wang X., Li S., Dhale N., Wu H., Van de Put M. L., Tiwari S., Vandenberghe W. G., Lv B. (2021). Phys. Rev. B.

[cit10] Kong Q., Qin R., Li D., Zhao H., Ren Y., Long L., Zheng L. (2019). RSC Adv..

[cit11] Zhou Y., Han S.-T. (2020). Science.

[cit12] Hedley L., Robertson N., Johansson J. O. (2017). Electrochim. Acta.

[cit13] Ohkoshi S.-i., Hashimoto K. (2002). The Electrochemical Society Interface.

[cit14] Ohkoshi S.-i., Iyoda T., Fujishima A., Hashimoto K. (1997). Phys. Rev. B.

[cit15] Tokoro H., Ohkoshi S.-i. (2011). Dalton Trans..

[cit16] Ohkoshi S.-i., Nakagawa K., Tomono K., Imoto K., Tsunobuchi Y., Tokoro H. (2010). Journal of the American Chemical Society.

[cit17] Zentkova M., Mihalik M. (2019). Crystals.

[cit18] Li W., Jafri H. M., Zhang C., Zhang Y., Zhang H., Huang H., Jiang S., Zhang G. (2020). J. Mater. Chem. A.

[cit19] Ma H., Gao W. X., Wang J. L., Wu T., Yuan G. L., Liu J. M., Liu Z. G. (2016). Adv. Electron. Mater..

[cit20] Liu H.-Y., Zhang H.-Y., Chen X.-G., Xiong R.-G. (2020). Journal of the American Chemical Society.

[cit21] Pajak Z., Czarnecki P., Szafranska B., Maluszynska H., Fojud Z. (2006). J. Chem. Phys..

[cit22] Hu Y., Zhu T., Guo Z., Popli H., Malissa H., Huang Y., An L., Li Z., Armstrong J. N., Boehme C., Vardeny Z. V., N'Diaye A. T., Zhou C., Wuttig M., Grossman J. C., Ren S. (2022). Nano Lett..

[cit23] Coates J. , Encyclopedia of Analytical Chemistry, 2006, 10.1002/9780470027318.a5606

[cit24] Lawes G., Srinivasan G. (2011). J. Phys. D: Appl. Phys..

[cit25] Liu M., Sun N. X. (2014). Philos. Trans. A Math. Phys. Eng. Sci..

[cit26] Jalouli A., Kilinc M., Marga A., Bian M., Thomay T., Petrou A., Zeng H. (2022). J. Chem. Phys..

[cit27] Takegahara K., Harima H. (2002). Phase Transitions.

