# Supplementary material for: Magnetoelectric interaction in molecular multiferroic nanocomposites
Source: RSC Adv. 2022 Aug 24;12(37):24050–4. doi: 10.1039/d2ra04060c (PMC9400656; doi:10.1039/d2ra04060c)
Supplement: RA-012-D2RA04060C-s001 [file RA-012-D2RA04060C-s001.pdf]

Electronic Supplementary Material (ESI) for RSC Advances.
This journal is © The Royal Society of Chemistry 2022

Supporting information

Alireza Jalouli[a], and Shenqiang Ren[a,b,c]

a. *Department of Mechanical and Aerospace Engineering, University at Buffalo, The State University of New York, Buffalo, New York, 14260, United States*
b. *Department of Chemistry, University at Buffalo, The State University of New York, Buffalo, New York, 14260, United States*
c. *Research and Education in Energy Environment & Water Institute, University at Buffalo, The State University of New York, Buffalo, New York, 14260, United States*

Sample preparation

To prepare PB powder crystals, the power of potassium hexacyanochromate (III) and vanadium (II) chloride are mixed with 3:2 weight ratio in water and is left in a centrifuge tube for one day to react completely. The product is washed for a few times and then centrifuged to remove the unreacted agents. The achieved substance with a dark blue color is dried for one day in a vacuum chamber at room temperature. The product is a dark blue powder of PB nanocrystals. Since the PB power is sensitive to oxidation, it is needed to kept in the glove box. To synthesis $ImClO_4$, equal molar amounts of imidazolium ($C_3N_2H_4$) with perchloric acid ($HClO_4$) are mixed. The solvent is dried slowly in several days to achieve a white transparent powder crystal. Different ratios of the synthesized crystals (PB and $ImClO_4$) were ground together and then pressed to have round palettes with 6mm diameter and about 1 mm thickness. To contact a thin copper wire to the top and bottom of the composite palette sample, silver epoxy was used. (Figure.S1(a)).

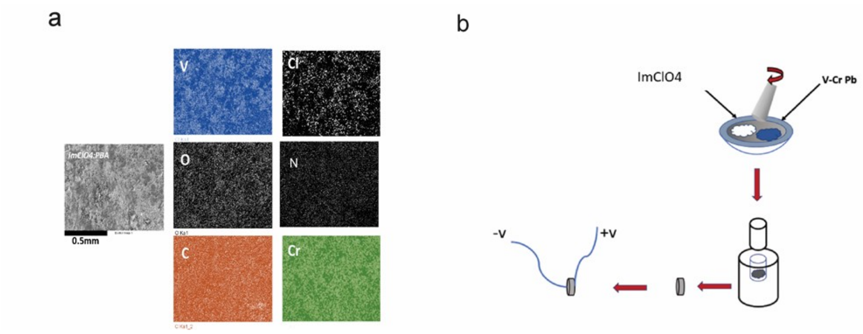

S1. (a) SEM/EDS mapping of the composite with Cl which is the representative of the. (b) The process of $ImClO_4$:PBA preparation.

Hysteresis measurement

The hysteresis measurements for three different ratios shows a weaker splitting for 2:1 (Fig. SI2(a)). To applied elect field to the two areas of the nanocomposite samples, two thin copper wire were attached to the areas by silver paste. Since $ImClO_4$ is an energetic material, during the experiment at room temperature, the dry nitrogen flow was provided to avoid over heating of the nanocomposite as a result of the applied high voltage. The current during the experiment was as low as 0.004 mA.

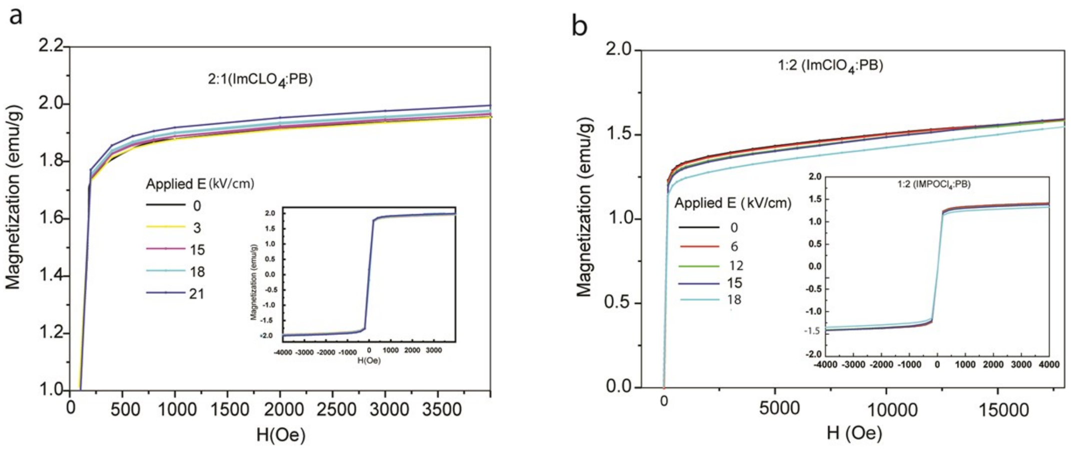

SI2. (a) hysteresis loop at room temperature for 2:1 at E=0, 3, 15, 18, and 21. (b) the hysteresis loop for 1:2

FMR measurement

FMR measurement in the absence of electric bias for 2:1 shows a similar trendline to 1:1 one. The FMR and integrated FMR signal at 4GHz (Fig. SI3(a), (b)) show the same magnitude of the shift (~5 Oe).

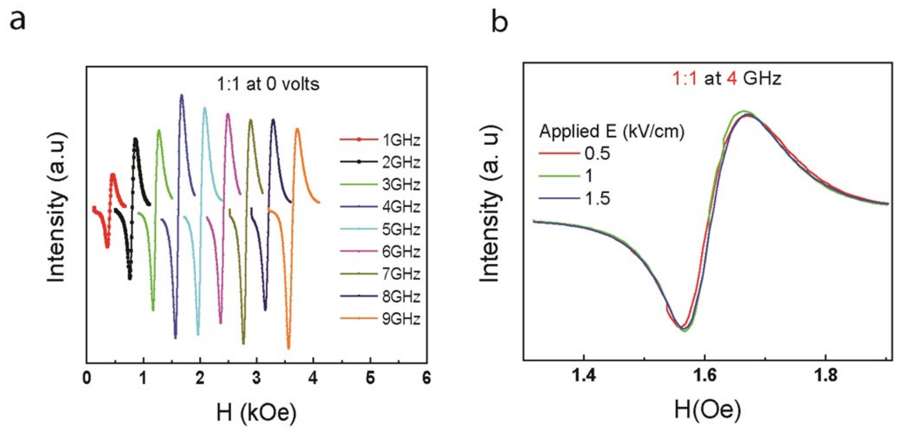

SI3. (a) FMR signal for various magnetic field in the absence of applied voltage. (b) Integrated FMR signal at 0.5, 1, and 1.5 kV/cm showing 5 Oe shift.
